# *Toxocara* Myopericarditis and Cardiac Magnetic Resonance Imaging in a Young Girl

**DOI:** 10.1155/2021/5526968

**Published:** 2021-05-03

**Authors:** Addison Gearhart, Timothy J. Savage, Thomas J. Sandora, Gabriella S. Lamb, Andrew J. Powell, Roger E. Breitbart

**Affiliations:** ^1^Department of Cardiology, Boston Children's Hospital, Boston, MA 02115, USA; ^2^Department of Pediatrics, Harvard Medical School, 25 Shattuck St, Boston, MA 02115, USA; ^3^Division of Infectious Diseases, Boston Children's Hospital, Boston, MA 02115, USA

## Abstract

Cardiac infection with *Toxocara* is rarely diagnosed, especially in children, and corresponding cardiac magnetic resonance imaging (CMR) has not been reported. We present a probable case, a 9-year-old girl with myopericarditis, eosinophilia, positive *Toxocara* serology, and CMR findings consistent with myocardial edema.

## 1. Background

Myopericarditis occurs as a result of acute inflammation of the pericardium and the underlying myocardium. A majority of cases are thought to be viral in origin, but other rarer infectious causes are known. It is important to establish the etiology as it may have implications for treatment and outcomes [1]. A subset of myopericarditis is associated with eosinophilia and may be related to drug hypersensitivity, autoimmunity, or parasitic infection [2].

Toxocariasis is a parasitic disease caused by *Toxocara canis* (dog roundworms) or *Toxocara cati* (cat roundworms). In the United States, *Toxocara* species are endemic, although the true prevalence of disease is likely under-recognized. Transmission follows human ingestion of eggs from contaminated dog or cat feces [3]. As many as 30% of young dogs deposit *Toxocara* eggs in their feces [4]. Children are considered to be particularly vulnerable due to poor hygiene. One survey found that nearly 14% of children >6 years of age were infected with *Toxocara* [5].

The pathogenesis of *Toxocara* disease is due to larval invasion into the vasculature and subsequent migration to one or more organs, leading to an inflammatory reaction [6]. Three clinical forms are described: visceral toxocariasis (visceral larva migrans) with involvement of the lungs, heart, and/or liver; ocular toxocariasis limited to the eye; and covert or common toxocariasis characterized by mild and nonspecific symptoms [3]. Cardiac involvement is rare. A systematic review of the literature identified only 24 reported cases of cardiac toxocariasis in the United States, 9 of which were pediatric, with rare mortality [7, 8]. The spectrum includes pericarditis, myocarditis, and Loeffler's endocarditis [3]. We describe a case of pediatric *Toxocara*-induced myopericarditis with positive *Toxocara* serology and cardiac magnetic resonance imaging (CMR) findings consistent with myocardial edema.

## 2. Case Presentation

**Figure 1:**
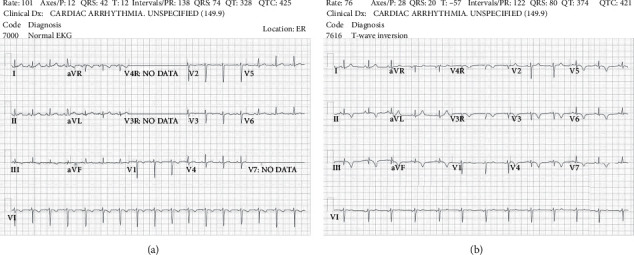
(a) The ECG was obtained on admission and shows a normal repolarization pattern. (b) The ECG represents the follow-up ECG at two weeks after presentation and shows diffuse T-wave inversion.

**Figure 2:**
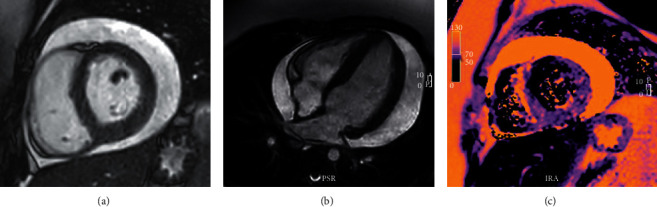
Cardiac MRI, cine short-axis (a) and 4-chamber (b) views showing a moderate-sized pericardial effusion. (c) Short-axis T2 map showing myocardial T2 is elevated (70 ms) consistent with diffuse edema.

A 9-year-old generally healthy girl with a history of a small muscular ventricular septal defect presented to the emergency room with chest pain and dyspnea. She had recovered from a recent upper respiratory infection and had also sustained minor trauma to the anterior chest wall in a fall a week prior. At presentation, she appeared well with temperature, 98.6 degrees Fahrenheit; heart rate, 104 beats per minute; respiratory rate, 28 breaths per minute; blood pressure, 113/66 mmHg; pulsus paradoxus, 8 mmHg; and normal pulse oximetry with minimal pulse amplitude variation on plethysmography. Her physical exam was notable only for mild tenderness over the sternum, with normal findings on cardiac auscultation. Chest radiography revealed a mildly enlarged cardiomediastinal silhouette and a focal opacity over the right middle lung possibly representing infection or pulmonary contusion. An electrocardiogram (ECG) was normal (Figure 1(a)). Echocardiography demonstrated a moderate-sized circumferential pericardial effusion, right atrial collapse, normal inflow and outflow variability across valves, normal biventricular systolic function, and no ventricular septal defect. Laboratory findings were notable for a leukocyte count of 12,000 cells/*µ*l with an elevated absolute eosinophil count of 1,570 cells/*µ*l (normal 40–190 cells/*µ*l), elevated C-reactive protein of 1.94 mg/dL (normal <0.5 mg/dL), elevated erythrocyte sedimentation rate of 45 mm/hr (normal 0–30 mm/hr), and elevated cardiac troponin T of 0.25 ng/mL (normal <0.1 ng/mL). CMR (Figure 2) showed an elevated myocardial global T2 value (70 ms), an elevated native myocardial global T1 value (1150 ms), increased left ventricular wall thickness, and a moderate-sized pericardial effusion. There was no myocardial late gadolinium enhancement.

**Figure 3:**
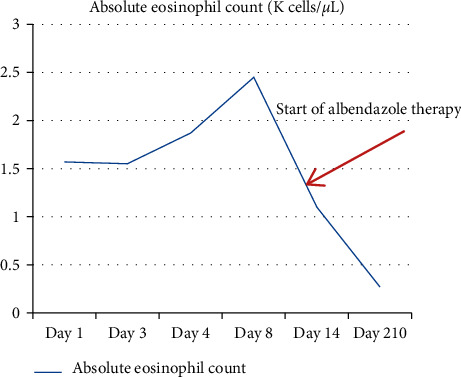
The shift of the serum eosinophil count from admission to follow-up. Normal range: 0.04–0.19 K cells/*µ*L.

The patient was admitted to the cardiology service. The working diagnosis was viral myopericarditis, but the differential diagnosis also included cardiac contusion, pulmonary contusion, and a range of etiologies for peripheral eosinophilia. Although some of the echocardiographic findings were consistent with tamponade physiology, the physical findings were not, and pericardiocentesis was not performed. Treatment was initiated with oral ibuprofen (400 mg every 8 hours) and colchicine (0.1 mg/kg daily) with resolution of chest pain, normalization of troponin and inflammatory markers, persistently normal ECGs, and reduction in the size of the pericardial effusion over three days. However, the peripheral eosinophilia increased (Figure 3). Consultation was obtained from the infectious diseases, allergy/immunology, and rheumatology services. Additional history was elicited, including pica and exposure to two dogs and a cat in the home. Further testing was recommended.

**Figure 4:**
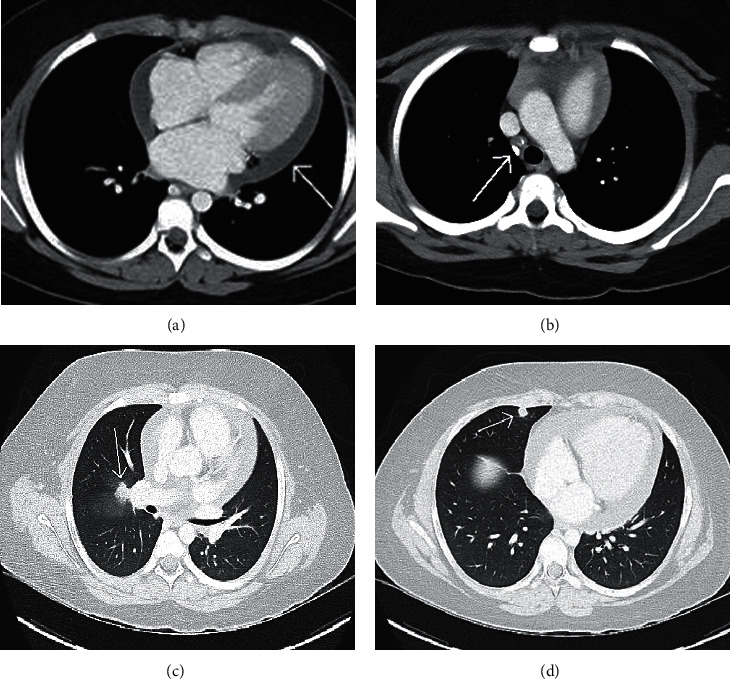
Axial CT images with contrast showing (a) large pericardial effusion and (b) calcified, but not enlarged right lower paratracheal region mediastinal nodes. (c) A 10 mm nodule at the right hilar region that has a mild spiculated appearance with adjacent ground glass opacities suggestive of hilar adenopathy versus pulmonary nodule. (d) Additional pulmonary nodule.

Chest computed tomography revealed multiple pulmonary nodules with adjacent ground glass opacities, consistent with vasculitis or infection (Figure 4). In the absence of systemic symptoms, an infectious workup was broadened, and a full rheumatologic evaluation and steroid initiation was postponed until the results of the infectious diseases workup were finalized. Microbiologic testing included negative PCRs for influenza, respiratory syncytial virus, parainfluenza virus, rhinovirus, and adenovirus. *Bartonella henselae* titers and a T-SPOT tuberculosis test were also negative. *Mycoplasma pneumoniae* IgM and IgG were elevated, but nasopharyngeal *Mycoplasma* PCR was negative. *Toxocara* serology was sent and was pending at discharge on hospital day 3.

At a two-week follow-up visit, the patient remained asymptomatic on ibuprofen and colchicine. Diffuse T-wave inversion appeared on serial ECGs, consistent with pericarditis (Figure 1(b)). Her absolute eosinophil count continued to rise to 2,450 cells/*µ*l. Twelve days into her illness, the *Toxocara* antibody resulted positive. An abdominal ultrasound examination was normal, without signs of liver involvement. A repeat echocardiogram showed a very small residual pericardial effusion. Ibuprofen and colchicine were discontinued, and treatment was initiated with a five-day course of oral albendazole (6 mg/kg/dose twice daily) and prednisolone (0.5 mg/kg/dose twice daily). At late follow-up there was a normal eosinophil count (Figure 3), and on echocardiogram, there was normal ventricular function and no pericardial effusion.

## 3. Discussion

The diagnosis of toxocariasis in this patient was based on positive serology and peripheral eosinophilia in the setting of likely exposure to *Toxocara* species. Imaging studies showed visceral involvement including the lung and heart. Liver involvement, reported on ultrasound in as many as 38% of cases, [9] was not found in this patient. Determination of myopericarditis was based on findings of pericardial effusion, elevated serum troponin, and CMR findings indicative of myocardial edema. Neither pericardial fluid sampling nor myocardial biopsy was undertaken in this patient, given her overall wellbeing, although these may contribute to the diagnosis of *Toxocara* myopericarditis in certain severe cases [3, 8, 10, 11].

This report is the first to document CMR abnormalities in a child with *Toxocara* infection. The value of CMR for evaluation of myocarditis in general in children is increasingly recognized [8]. In our patient, findings of elevated global T1 and T2 values fulfilled the 2018 Lake Louise criteria for the diagnosis of acute myocarditis [10]. Early identification of myopericarditis led to a focused infectious workup and ultimately early identification of *Toxocara* and treatment.

There are no guidelines for treatment of *Toxocara* myopericarditis. A five-day course of albendazole is the current standard for visceral larva migrans with the recommendation of adjunct corticosteroids for signs of systemic inflammation [11]. Case reports include remission with antihelminthics or corticosteroids alone or in combination, with early steroid therapy associated with better outcomes [7, 12]. The duration and dosing of this combination therapy has yet to be defined. Shorter courses have been reported as effective, although recurrences have also been documented [13]. Some authors have advocated for longer treatment duration with cardiac involvement [7]. We elected to treat for just five days with albendazole and prednisolone, given the early diagnosis and relatively benign presentation without ventricular dysfunction, arrhythmia, or systemic symptoms and given the rapid initial response to ibuprofen and colchicine. Serologic titers may remain positive for years following adequate treatment; thus, clinical improvement and normalization of eosinophil count are the recommended parameters for monitoring response to therapy. A second course of antihelminthics and steroids may be necessary to achieve this.

## 4. Conclusion

*Toxocara* infection is an underappreciated cause of morbidity that should be considered in children presenting with myopericarditis associated with peripheral eosinophilia. CMR may aid in the diagnosis of myopericarditis. Treatment has included albendazole in combination with corticosteroids, with treatment length determined through monitoring for normalization of eosinophilia and clinical improvement.

## Data Availability

The data used to support the findings of this report are included within the article.
